# Supplementary material for: Terpene Glycosides from Sanguisorba officinalis and Their Anti-Inflammatory Effects
Source: Molecules. 2019 Aug 10;24(16):2906. doi: 10.3390/molecules24162906 (PMC6720167; doi:10.3390/molecules24162906)
Supplement: Supplementary file 1 [file molecules-24-02906-s001.pdf]

Supporting information

Two new terpene glycosides from *Sanguisorba officinalis* and their anti-inflammatory effects

Dale Guo[1], Jinfeng Chen[1], Lu Tan[1], Mengying Jin[1], Feng Ju[1], Zhixing Cao[1], Fang Deng[1], Lina Wang[1, 2, *], Yucheng Gu[3], Yun Deng[1, *]

1.The Ministry of Education Key Laboratory of Standardization of Chinese Herbal Medicine, State Key Laboratory, Breeding Base of Systematic Research Development and Utilization of Chinese Medicine Resources, School of Pharmacy, Chengdu University of Traditional Chinese Medicine, Chengdu 611137, P R China

2. School of Nursing, Sichuan Tianyi College, Mianzhu 618200, P R China

3. Syngenta Jealott's Hill International Research Centre, Berkshire RG42 6EY, UK

*Corresponding author. Tel/Fax: +86 028 61800232.

E-mail address: wanglina1871@163.com (Lina Wang)

dengyun@cdutcm.edu.cn (Yun Deng)

Tables of Contents

S1. HRESIMS spectrum of **1**

S2. ^{1}H NMR spectrum (400 MHz, CD_3OD) of **1**

S3. ^{13}C NMR spectrum (100 MHz, CD_3OD) of **1**

S4. ^{1}H-^{1}H COSY spectrum (CD_3OD) of **1**

S5. HSQC spectrum (CD_3OD) of **1**

S6. HMBC spectrum (CD_3OD) of **1**

S7. NOESY spectrum (CD_3OD) of **1**

S8. HRESIMS spectrum of **2**

S9. ^{1}H NMR spectrum (400 MHz, CD_3OD) of **2**

S10. ^{13}C NMR spectrum (100 MHz, CD_3OD) of **2**

S11.^{1}H-^{1}H COSY spectrum (CD_3OD) of **2**

S12. HMQC spectrum (CD_3OD) of **2**

S13. HMBC spectrum (CD_3OD) of **2**

S14. NOESY spectrum (CD_3OD) of **2**

S15. The HPLC profiles of stand monosaccharides and compounds **1-2** after acid hydrolysis.

S16. The HPLC profiles of the derivatives of stand monosaccharides and compounds **1-2** after acid hydrolysis.

S17. RAW264.7 cell viability assay of compounds **1**-**4**.

S18. Effect of dexamethasone (DXMS) and compounds **1**-**4** on the macrophage's distribution of zebrafish.

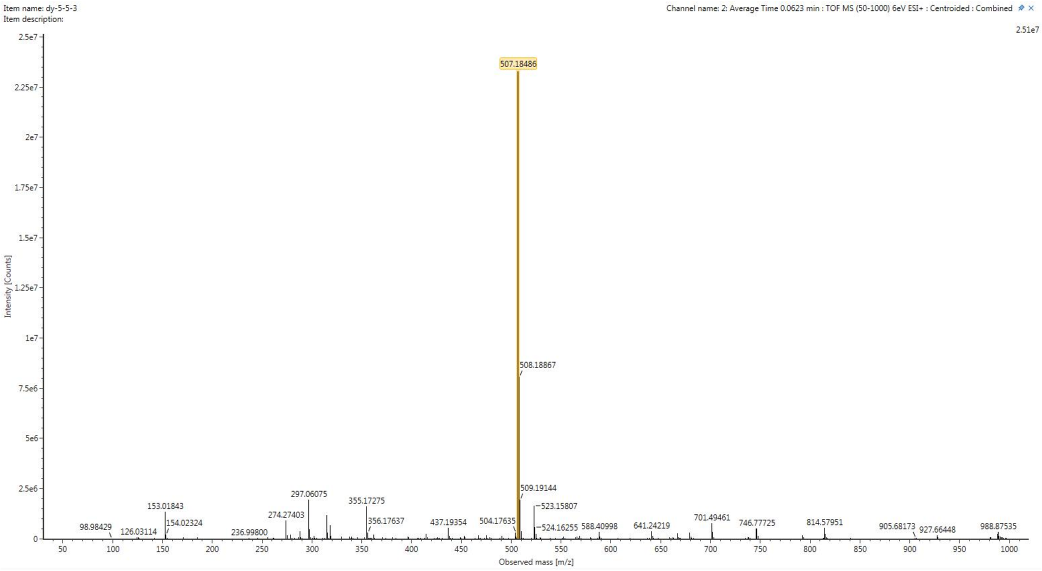

S1. HRESIMS spectrum of **1**

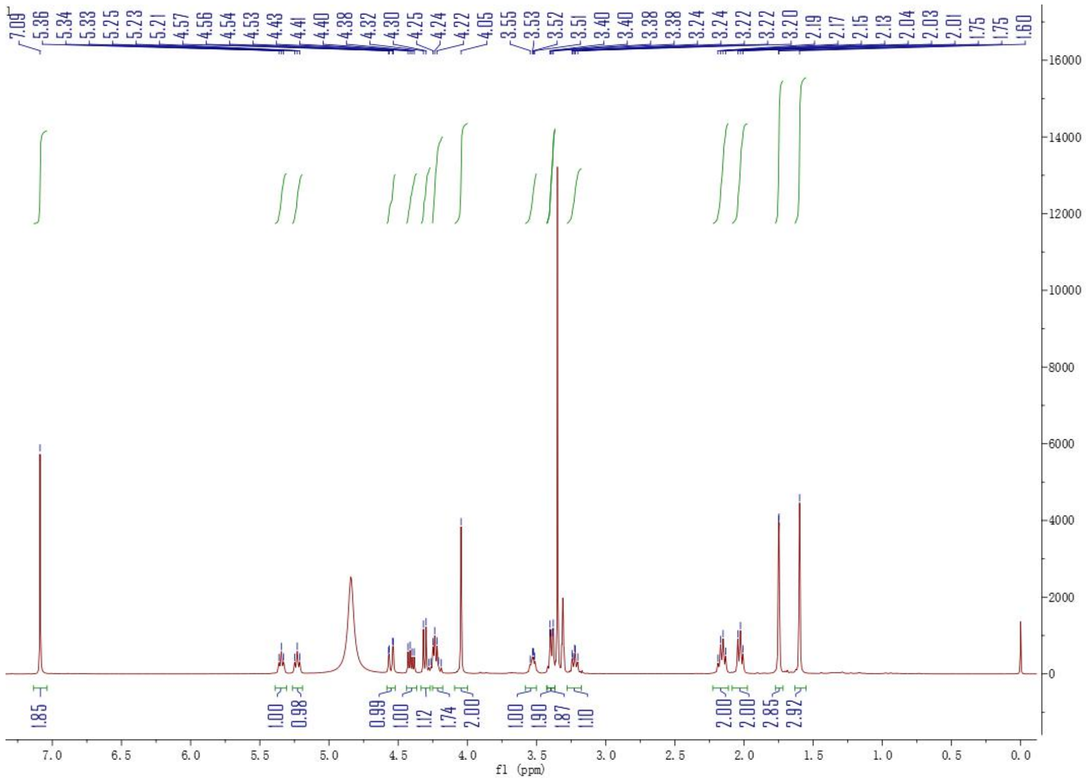

S2. ^{1}H NMR spectrum (400 MHz, CD_3OD) of **1**

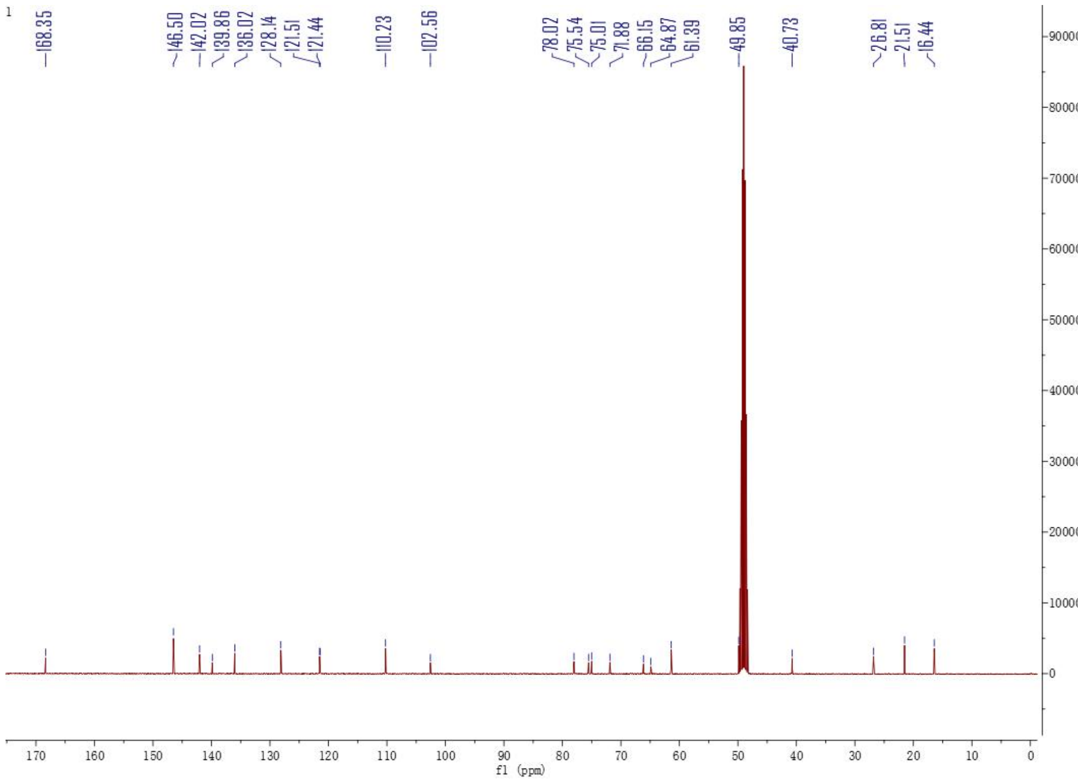

S3. ^{13}C NMR spectrum (100 MHz, CD_3OD) of **1**

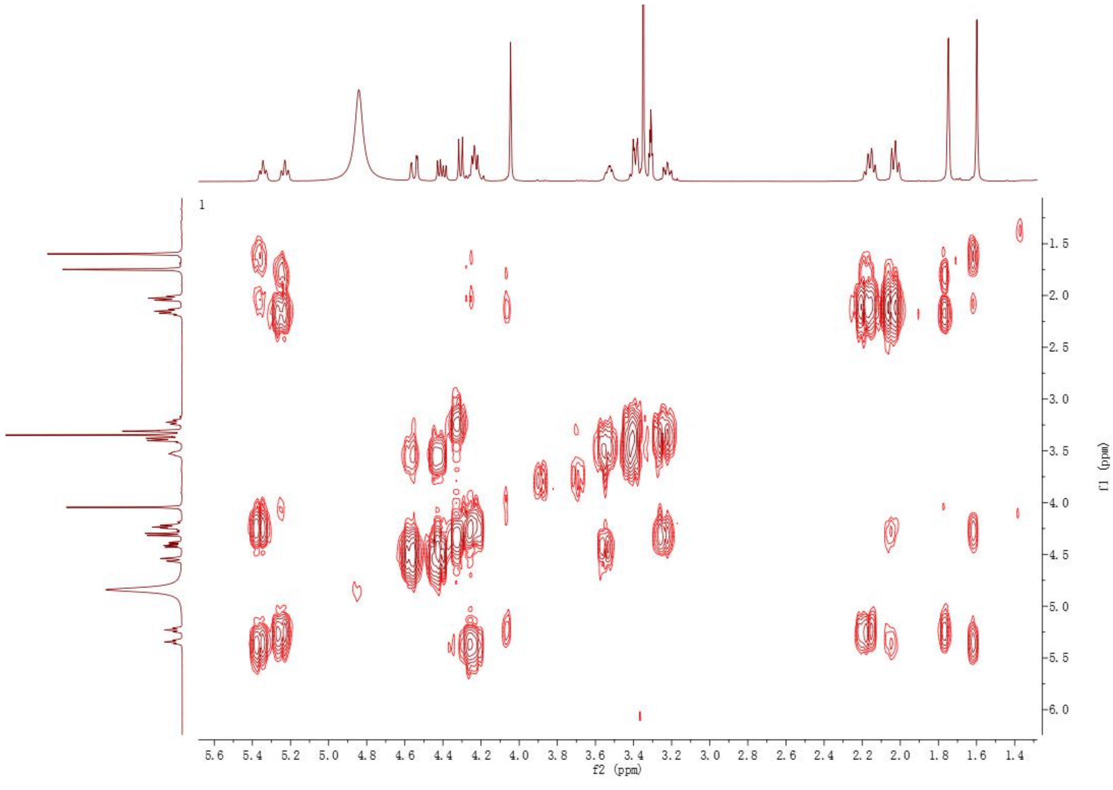

S4. ^{1}H-^{1}H COSY spectrum (CD_3OD) of **1**

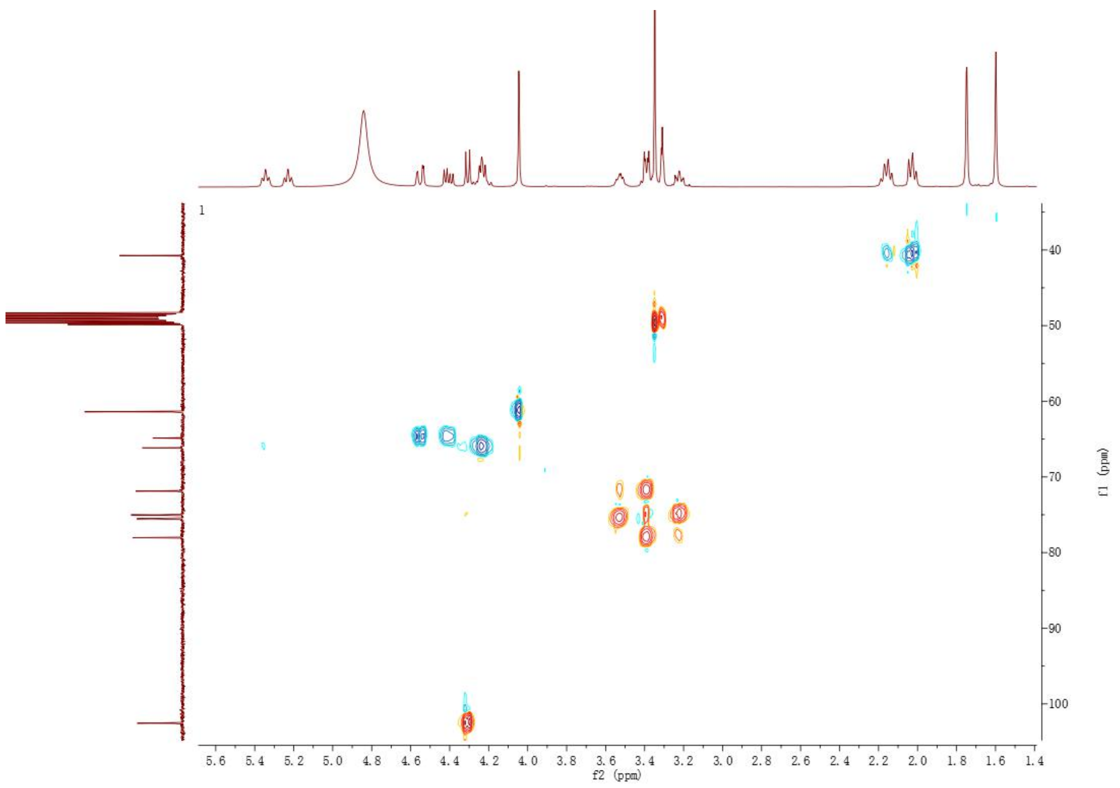

S5. HSQC spectrum (CD_3OD) of **1**

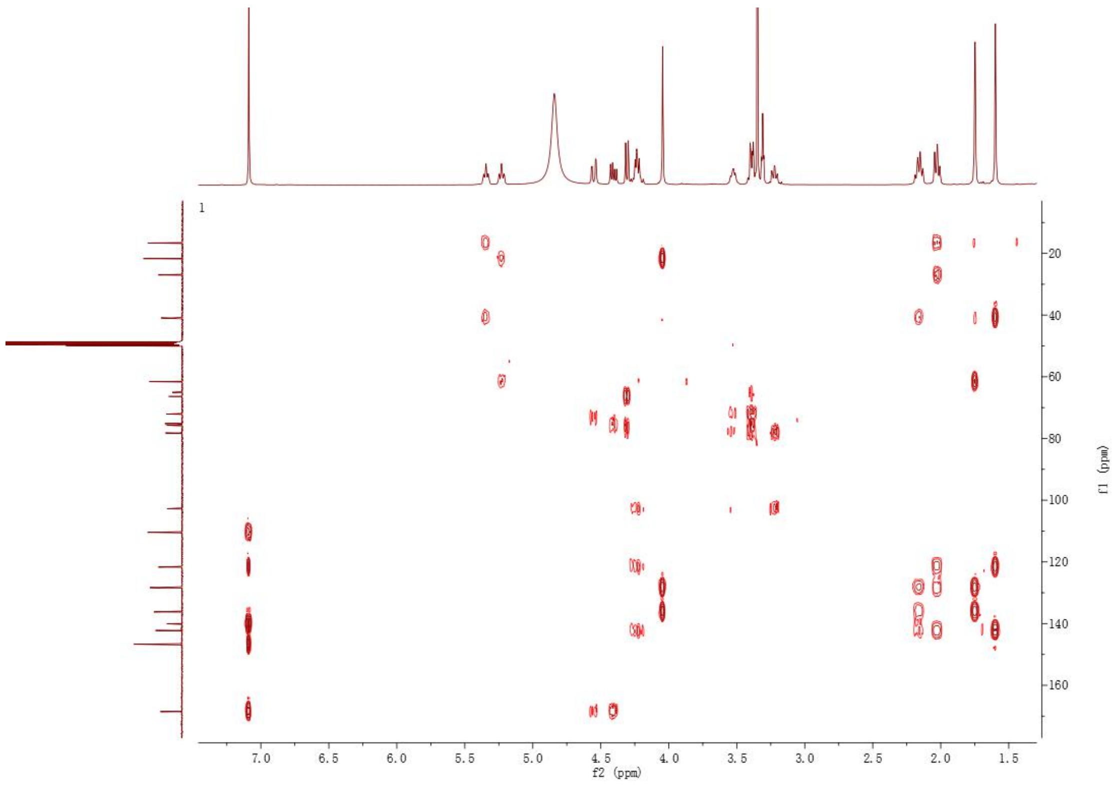

S6. HMBC spectrum (CD_3OD) of **1**

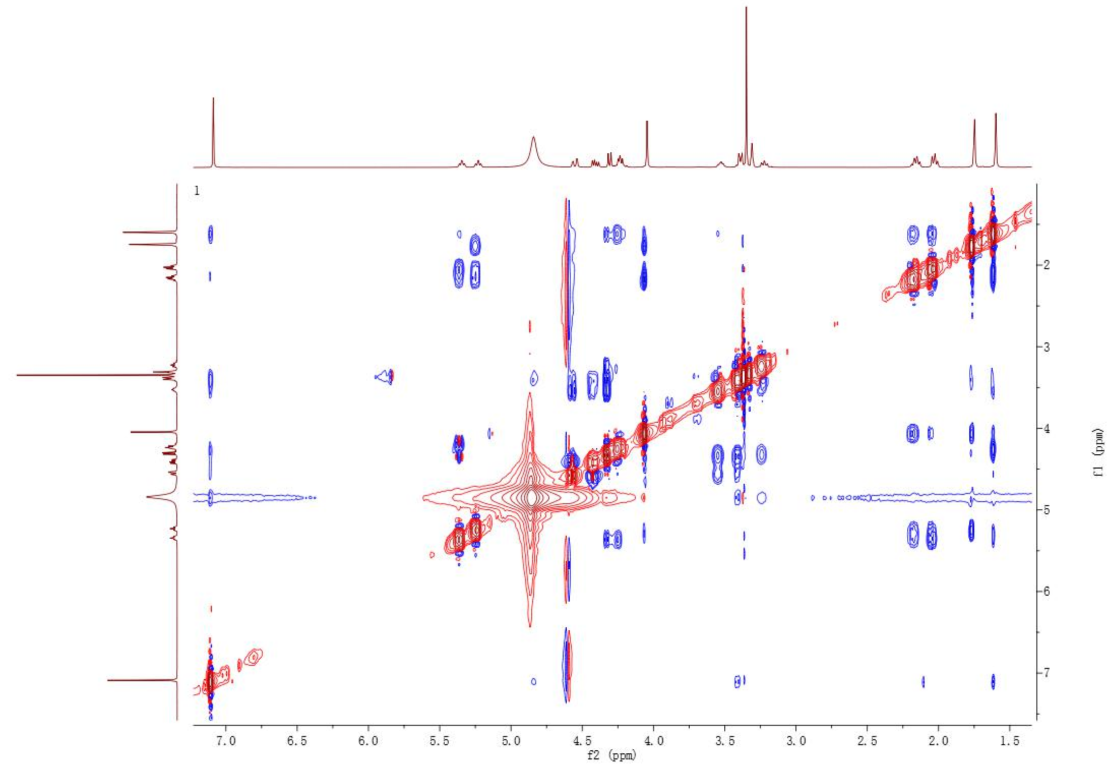

S7. NOESY spectrum (CD_3OD) of **1**

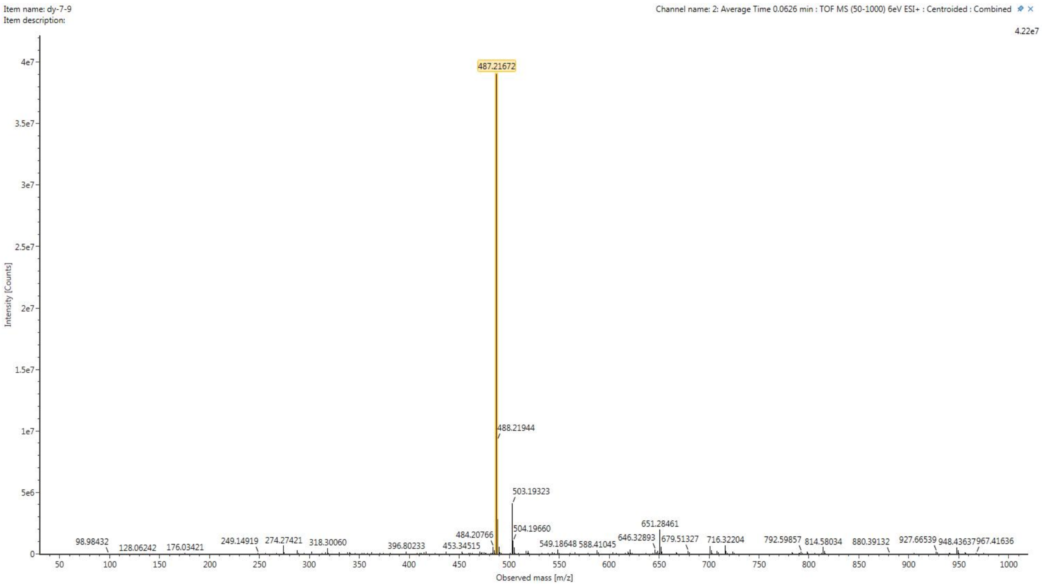

S8. HRESIMS spectrum of **2**

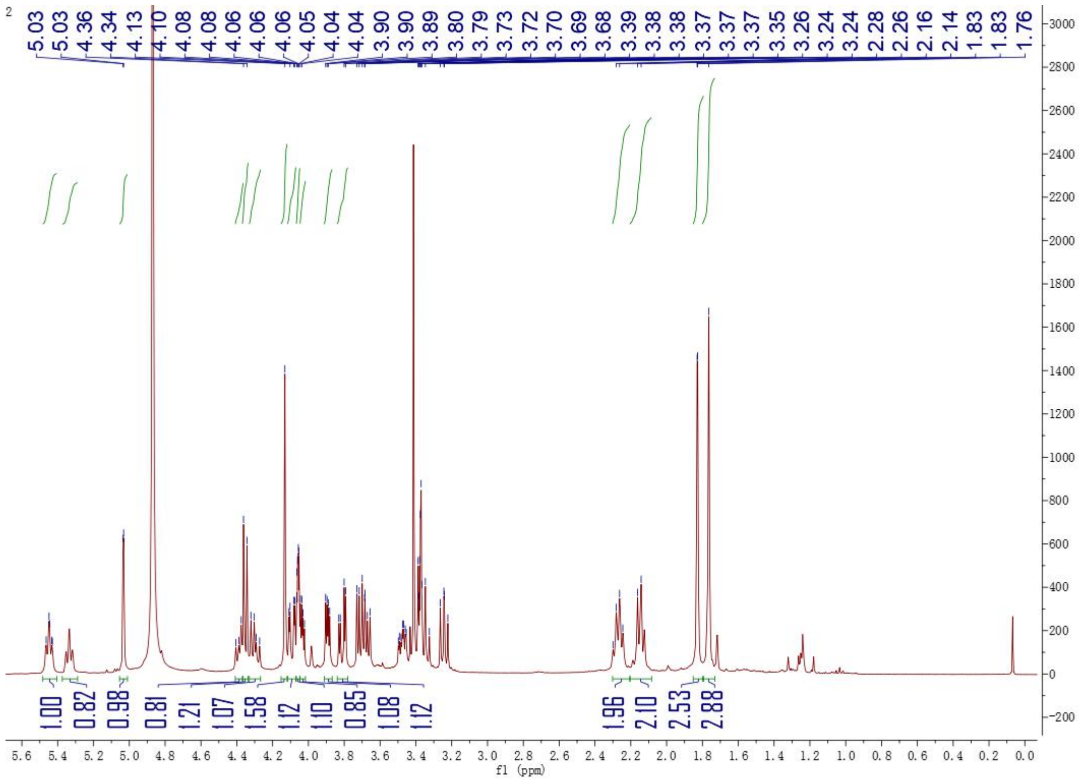

S9. ^{1}H NMR spectrum (400 MHz, CD_3OD) of **2**

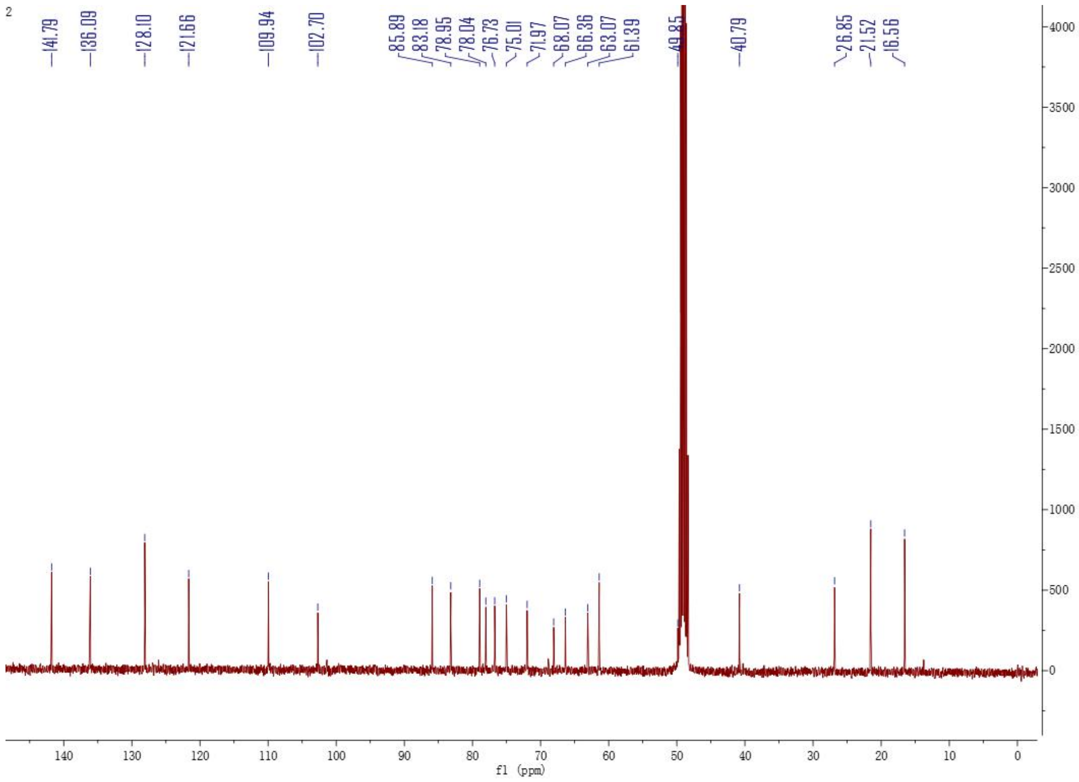

S10. ^{13}C NMR spectrum (100 MHz, CD_3OD) of **2**

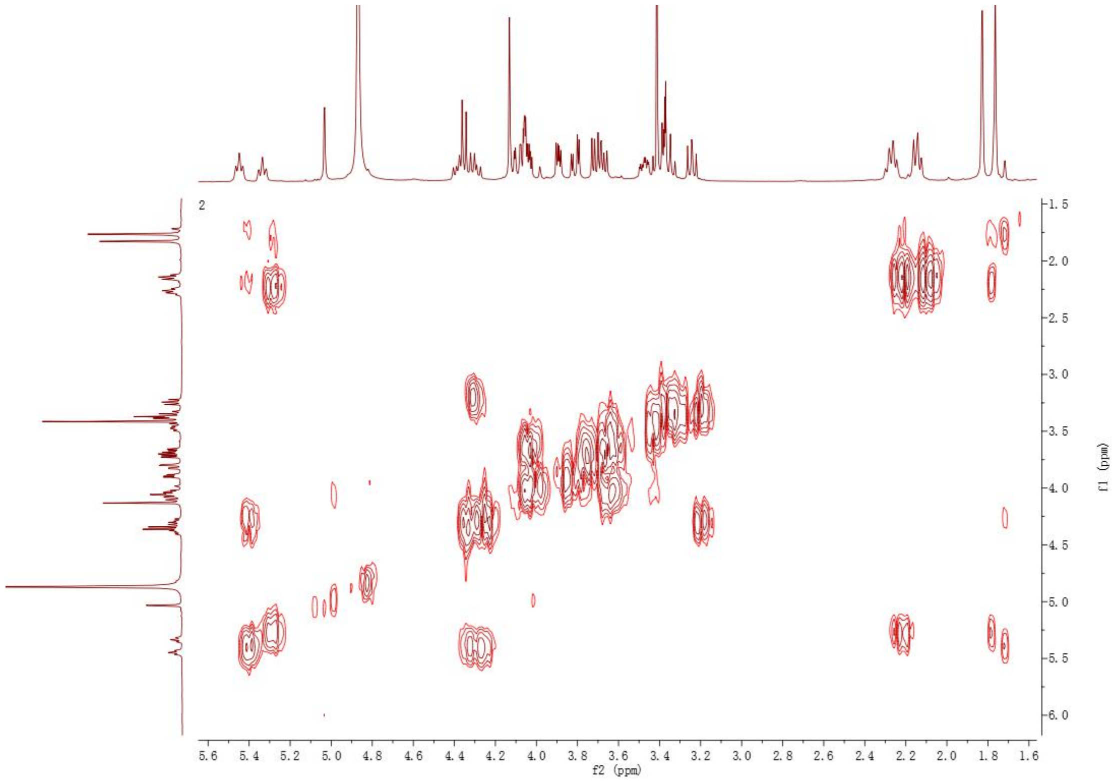

S11.^{1}H-^{1}H COSY spectrum (CD_3OD) of **2**

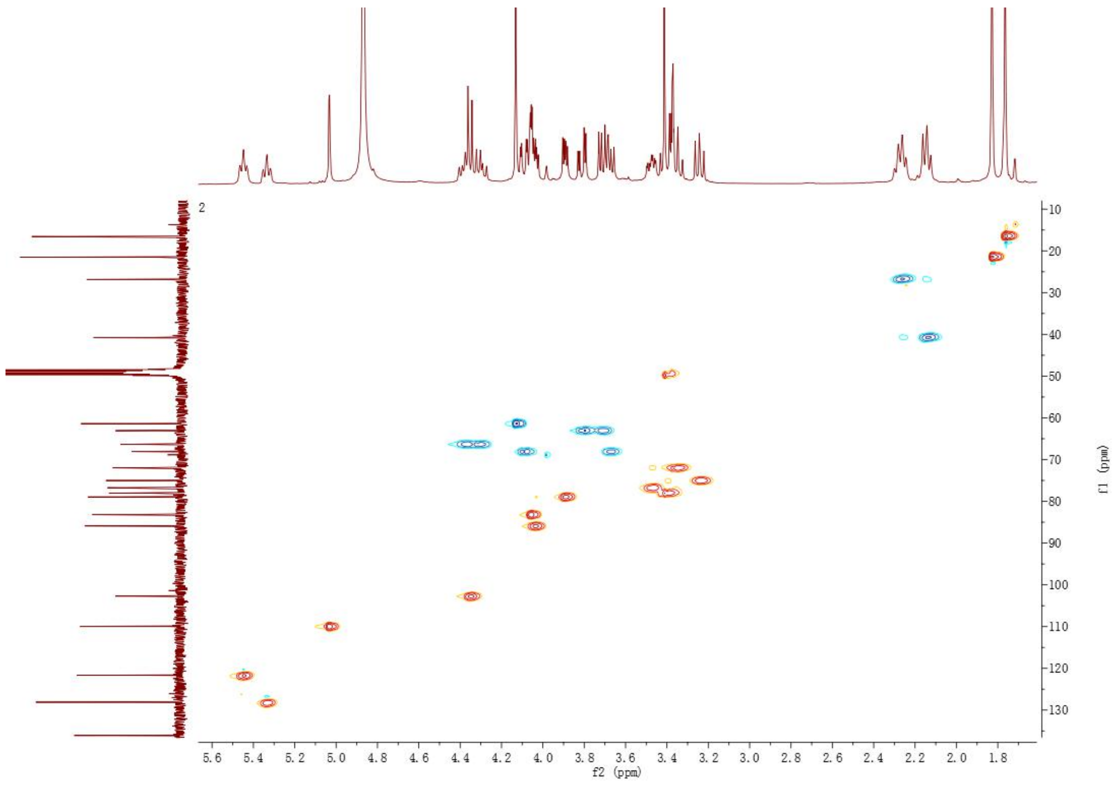

S12. HMQC spectrum (CD_3OD) of **2**

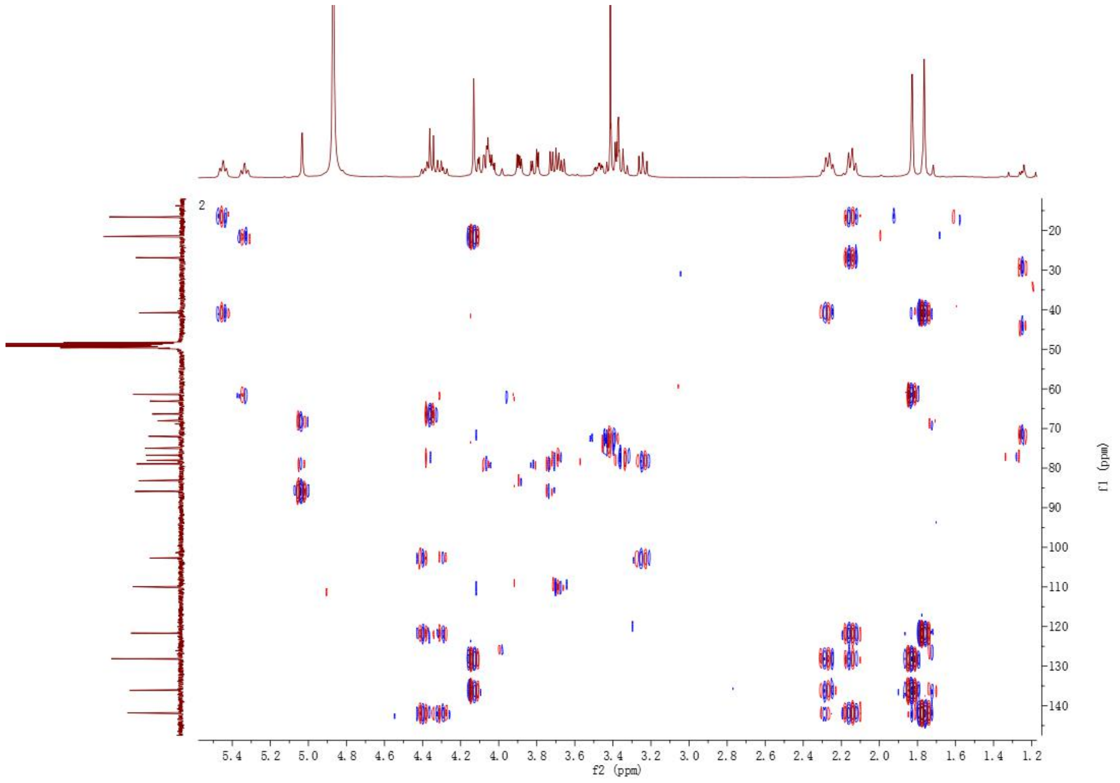

S13. HMBC spectrum (CD_3OD) of **2**

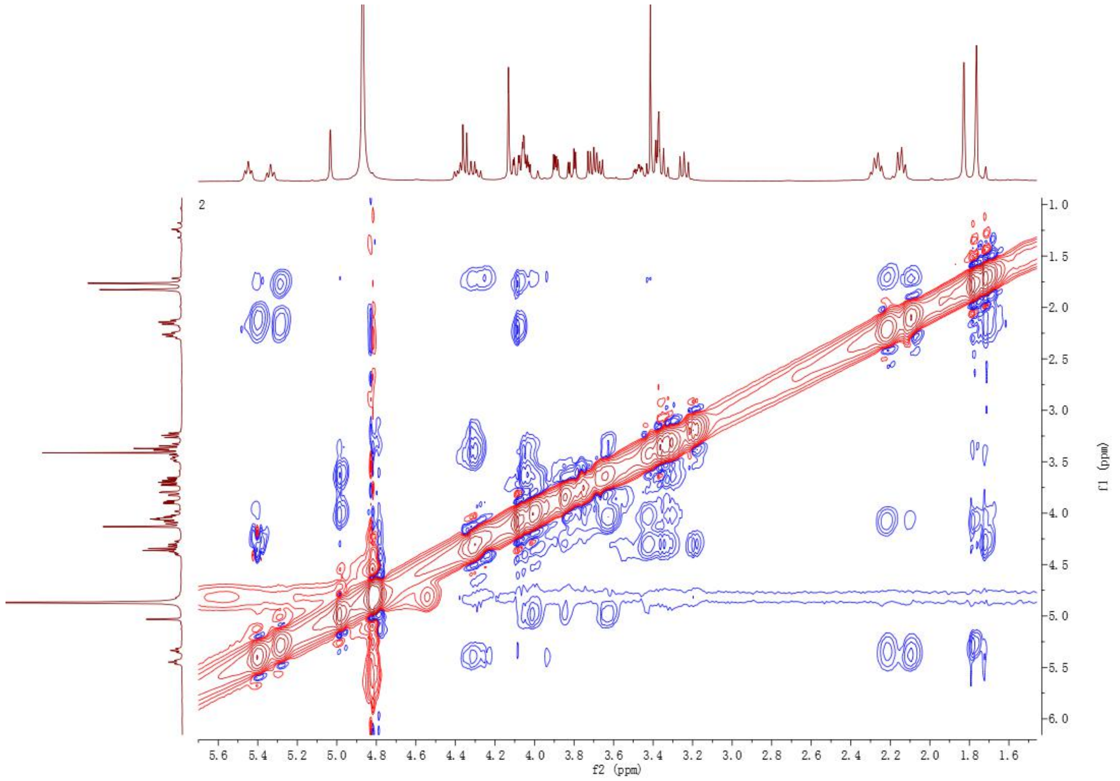

S14. NOESY spectrum (CD_3OD) of **2**

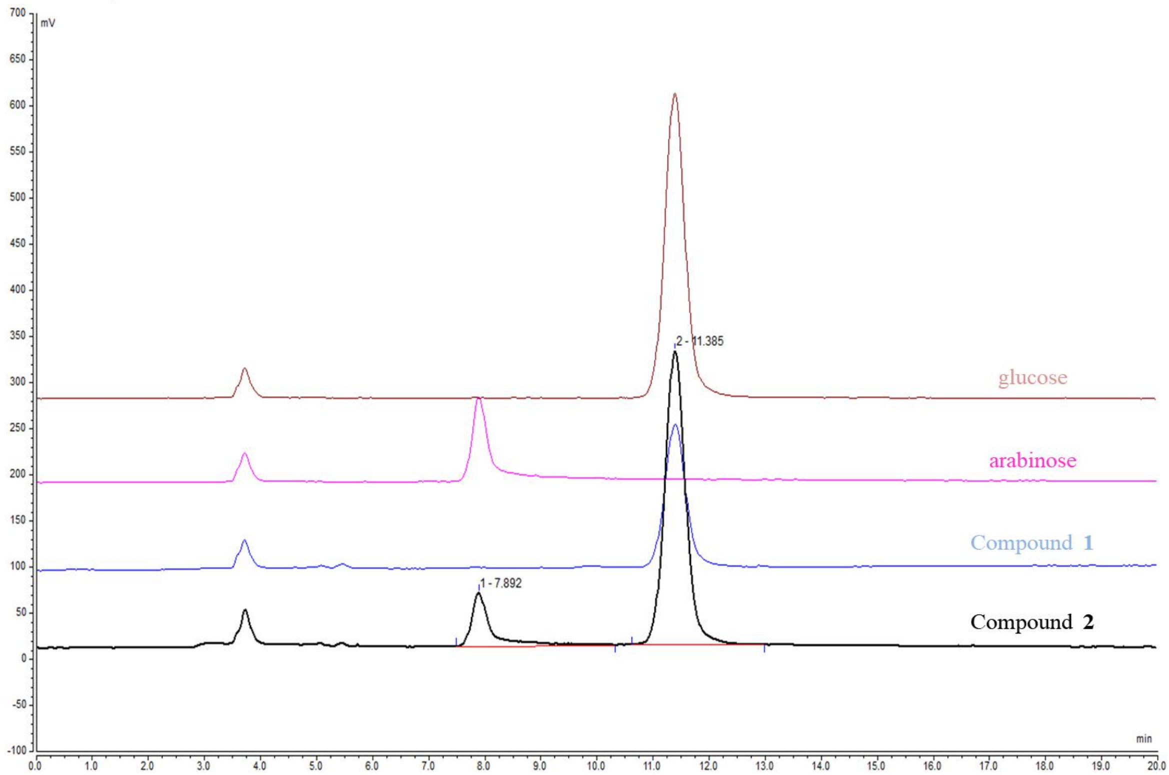

S15. The HPLC profiles of stand monosaccharides and compounds **1-2** after acid hydrolysis.

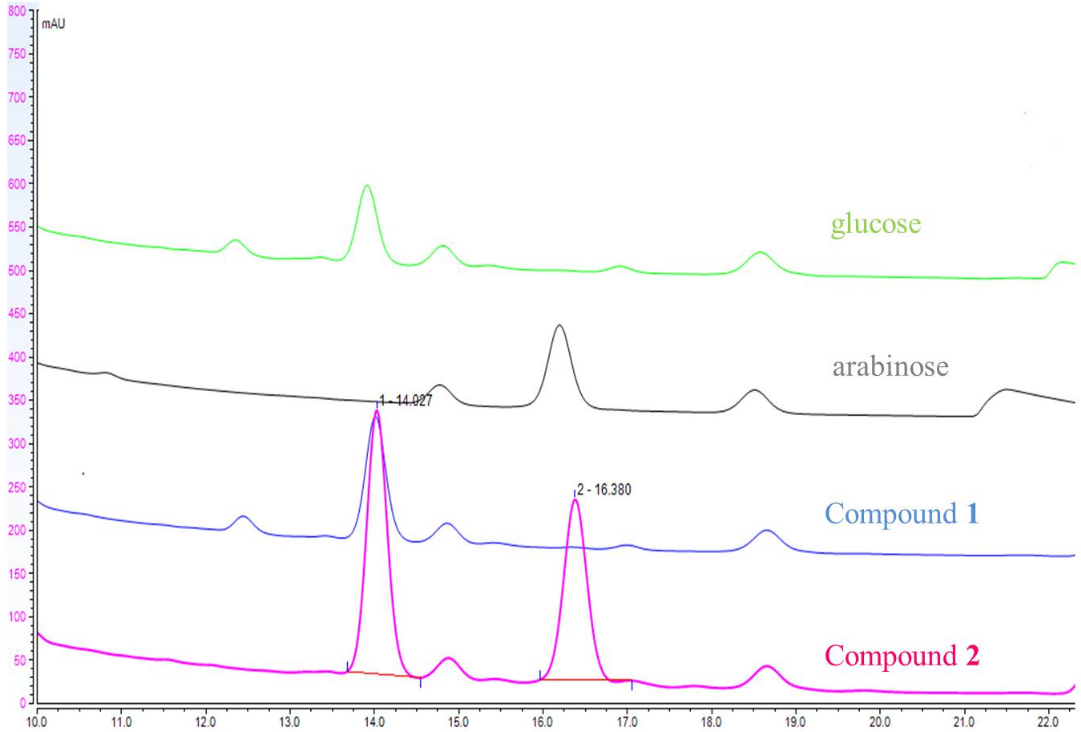

S16. The HPLC profiles of the derivatives of stand monosaccharides and compounds **1-2** after acid hydrolysis.

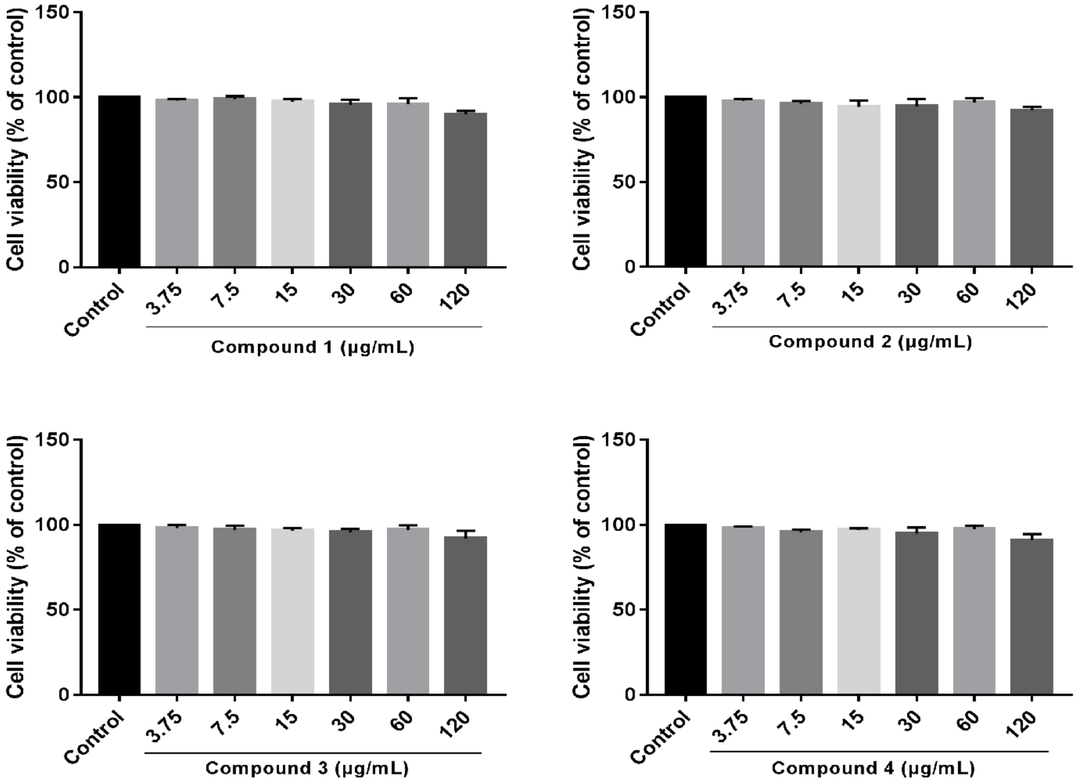

S17. RAW264.7 cell viability assay of compounds **1-4**

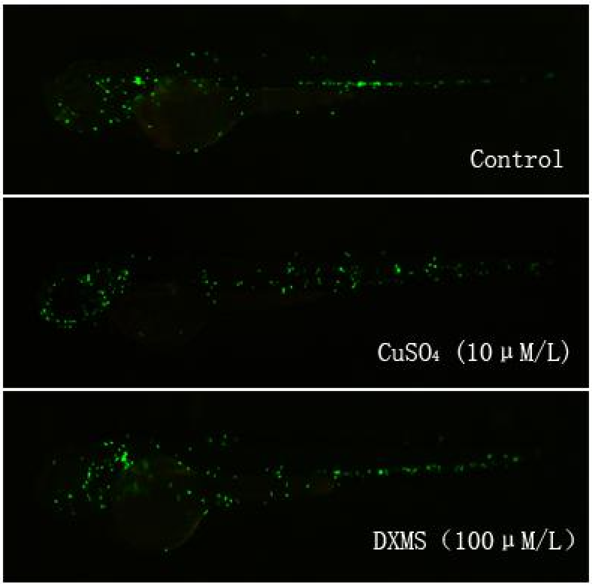

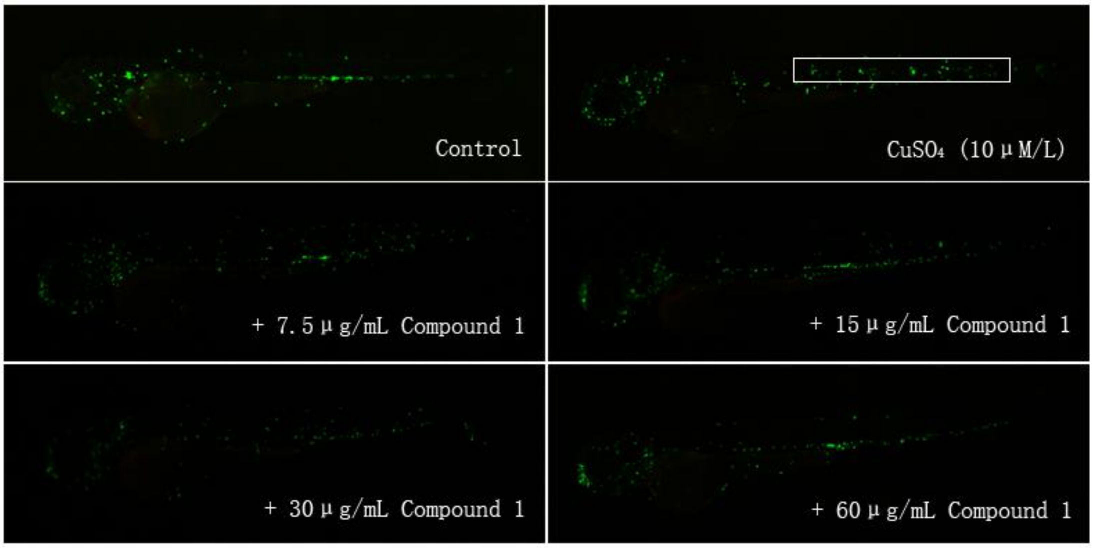
Control
CuSO$_4$ (10 μM/L)
+ 7.5 μg/mL Compound 1
+ 15 μg/mL Compound 1
+ 30 μg/mL Compound 1
+ 60 μg/mL Compound 1

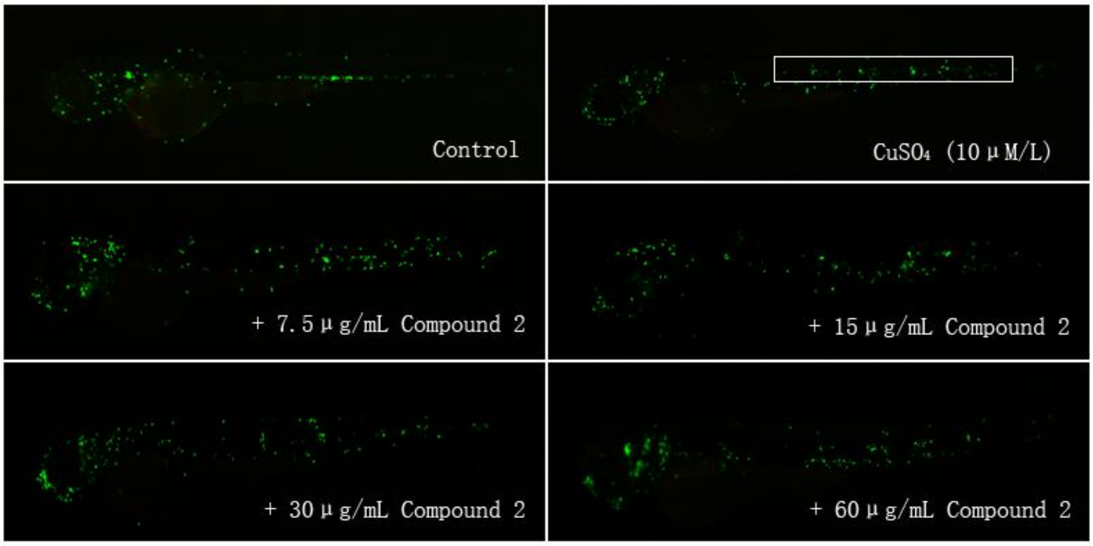
Control
CuSO$_4$ (10 μM/L)
+ 7.5 μg/mL Compound 2
+ 15 μg/mL Compound 2
+ 30 μg/mL Compound 2
+ 60 μg/mL Compound 2

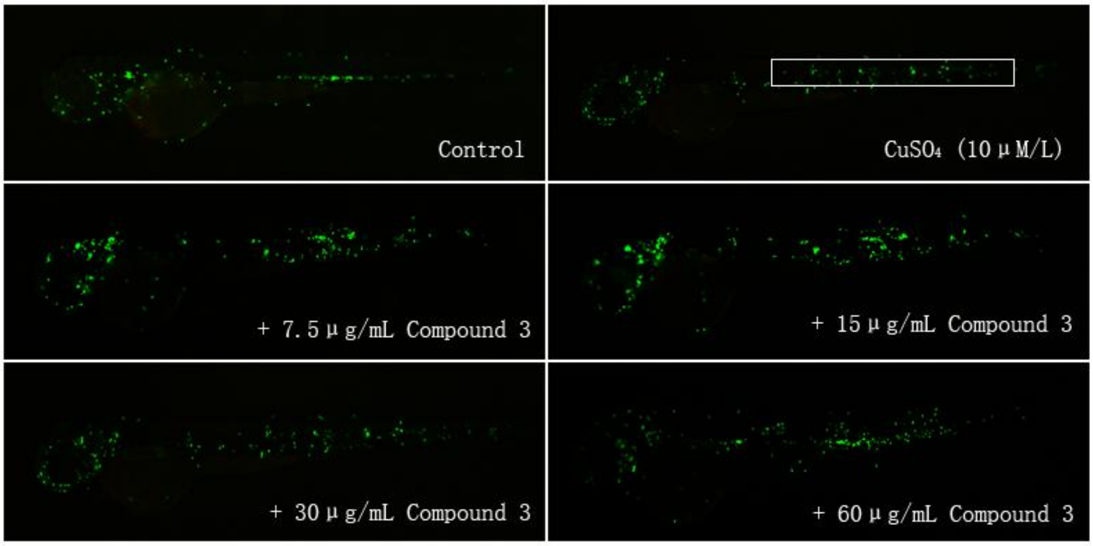
Control
CuSO$_4$ (10 μM/L)
+ 7.5 μg/mL Compound 3
+ 15 μg/mL Compound 3
+ 30 μg/mL Compound 3
+ 60 μg/mL Compound 3

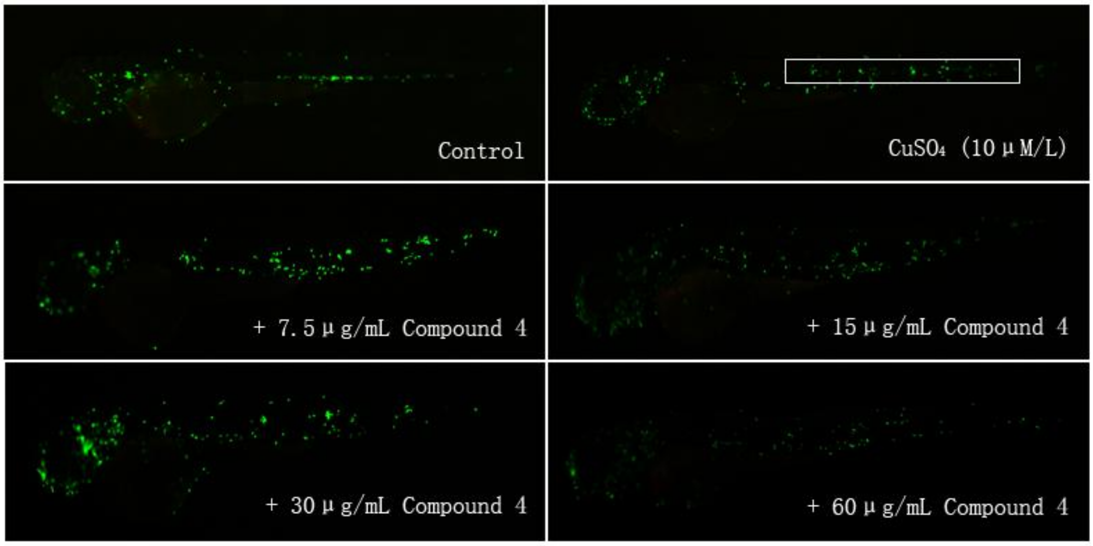

S18. Effect of dexamethasone and compounds **1**-**4** on the macrophage's distribution of zebrafish.
